# Supplementary material for: Identification of signaling pathways, matrix-digestion enzymes, and motility components controlling Vibrio cholerae biofilm dispersal
Source: Proc Natl Acad Sci U S A. 2020 Dec 7;117(51):32639–47. doi: 10.1073/pnas.2021166117 (PMC7768729; doi:10.1073/pnas.2021166117)
Supplement: Supplementary File [file pnas.2021166117.sapp.pdf]

PNAS
www.pnas.org

Supplementary Information for

Identification of signaling pathways, matrix-digestion enzymes, and motility components controlling *Vibrio cholerae* biofilm dispersal

Andrew A. Bridges[1,2], Chenyi Fei[1], Bonnie L. Bassler[1,2*]
[1]Department of Molecular Biology, Princeton University, Princeton, NJ 08544, USA
[2]The Howard Hughes Medical Institute, Chevy Chase, MD 20815, USA.

*Corresponding author: Bonnie L. Bassler
Email: bbassler@princeton.edu

This PDF file includes:

Supplementary text
Figures S1 to S4
Tables S1 to S2
Legends for Movie S1
SI References

Other supplementary materials for this manuscript include the following:

Movie S1

www.pnas.org/cgi/doi/10.1073/pnas.2021166117

Supplementary Information Text

DbfS is not equivalent to PhoQ

In *Escherichia coli*, low Mg^{2+} and cationic peptides activate PhoQ kinase activity (1). Sequence alignment of the DbfS sensory domain with that from PhoQ of *E. coli*, *Salmonella enterica*, and *Pseudomonas aeruginosa* revealed that DbfS lacks all of the key residues involved in Mg^{2+} binding (Fig. S2A) (2). To test if Mg^{2+} alters DfbS activity, we measured the *V. cholerae* biofilm lifecycle in response to low Mg^{2+} conditions in WT *V. cholerae* and in the Δ*dbfR* mutant. If, analogous to PhoQ, DfbS kinase activity is activated by low Mg^{2+}, when Mg^{2+} is limiting, WT *V. cholerae* should exhibit an altered biofilm dispersal phenotype while the Δ*dbfR* mutant would be impervious to Mg^{2+} changes (1). Fig. S2B shows that Mg^{2+} limitation does indeed inhibit *V. cholerae* biofilm dispersal, however, inhibition occurs in *both* the WT and the Δ*dbfR* strains. Mg^{2+} limitation did not alter *vpsL-lux* expression in either strain (Fig. S2C). Thus, Mg^{2+} does not control DfbS activity. We obtained the same results following exogenous addition of the cationic peptide C18G (Fig. S2D). Together, these results demonstrate that DfbS does not respond to the ligands that control PhoQ activity.

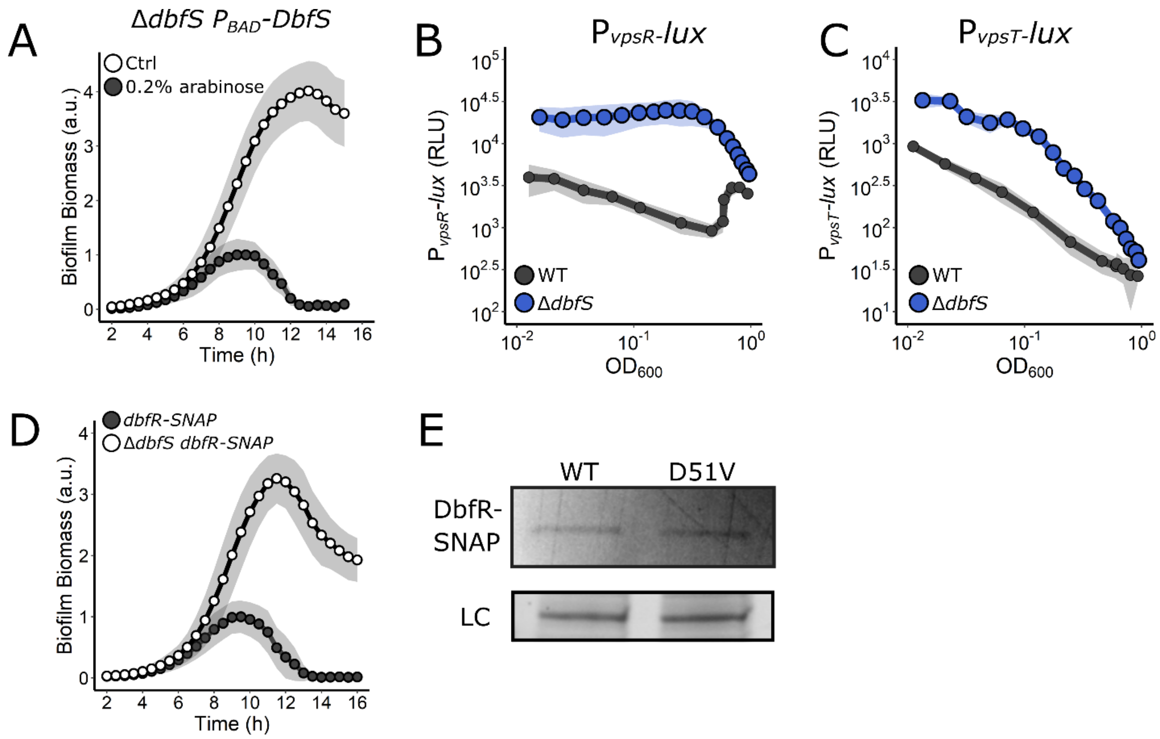

Fig. S1. Complementation, functional tagging, and mutagenesis of the DbfS-DbfR two-component system. (A) Quantitation of biofilm biomass over time measured by time-lapse microscopy for the Δ*dbfS* P_{BAD}-*dbfS* strain following addition of water (Ctrl) or 0.2% arabinose. (B) P_{vpsR}-*lux* and (C) P_{vpsT}-*lux* output for WT and the Δ*dbfS* strain over the growth curve. (D) As in A for SNAP-tagged DbfR in the WT and Δ*dbfS* strains. (E) Top panel: representative in-gel SDS-PAGE fluorescence following electrophoresis of *V. cholerae* cell lysates containing WT DbfS-SNAP or DbfS^{D51V}-SNAP that had been incubated with SNAP-Cell TMR Star. Bottom panel: Coomassie stained loading control (LC). For all biofilm measurements, *N* = 3 biological and *N* = 3 technical replicates, ± SD (shaded). a.u., arbitrary unit. For *lux* measurements, *N* = 3 biological replicates, ± SD (shaded). RLU, relative light units.

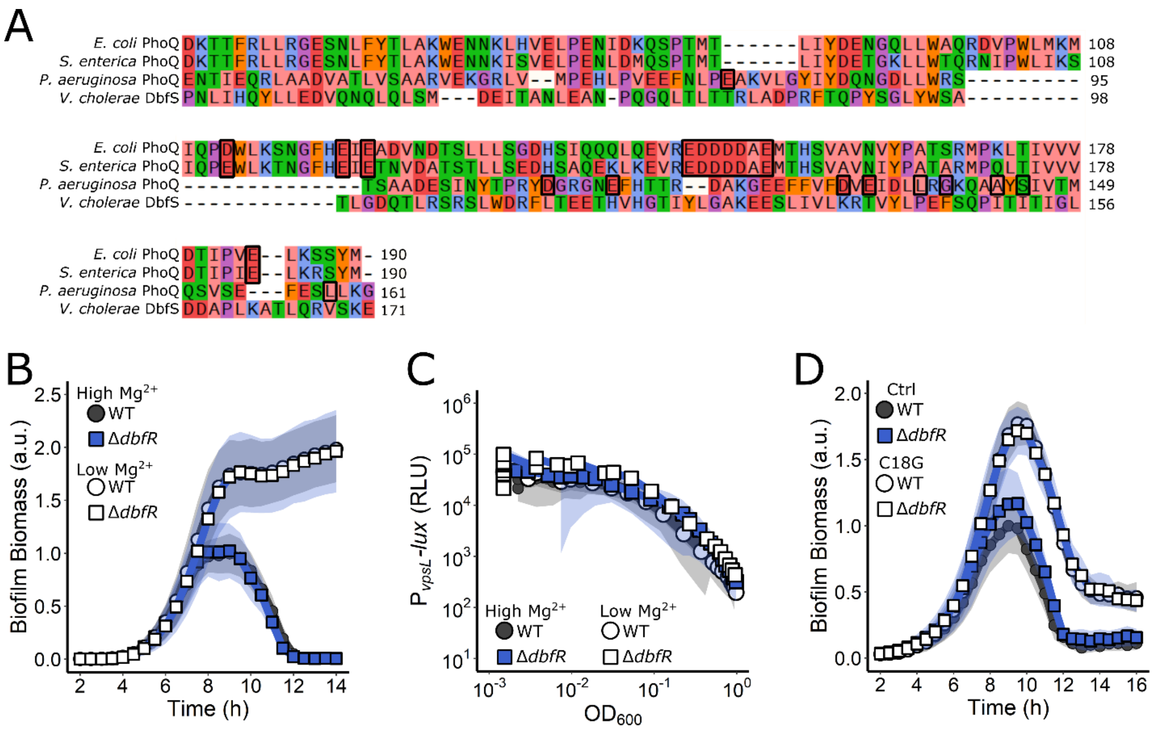

Fig. S2. DbfS is not functionally equivalent to PhoQ. (A) Alignment of the sensory domains of PhoQ from *E. coli*, *S. enterica*, and *P. aeruginosa* against that of *V. cholerae* DbfS. Black boxes indicate residues involved in Mg^{2+} binding in PhoQ. (B) Quantitation of biofilm biomass over time measured by time-lapse microscopy in high magnesium (10 mM) and limiting magnesium (10 µM) conditions for WT *V. cholerae* and the Δ*dbfR* strain. (C) The corresponding P_{vpsL}-*lux* outputs for strains and growth conditions in B over the growth curve. (D) As in B except following the addition of water or 5 µg/mL C18G. In all cases, *N* = 3 biological and *N* = 3 technical replicates, ± SD (shaded). a.u., arbitrary unit. For *vpsL-lux* measurements, *N* = 3 biological replicates, ± SD (shaded). RLU, relative light units.

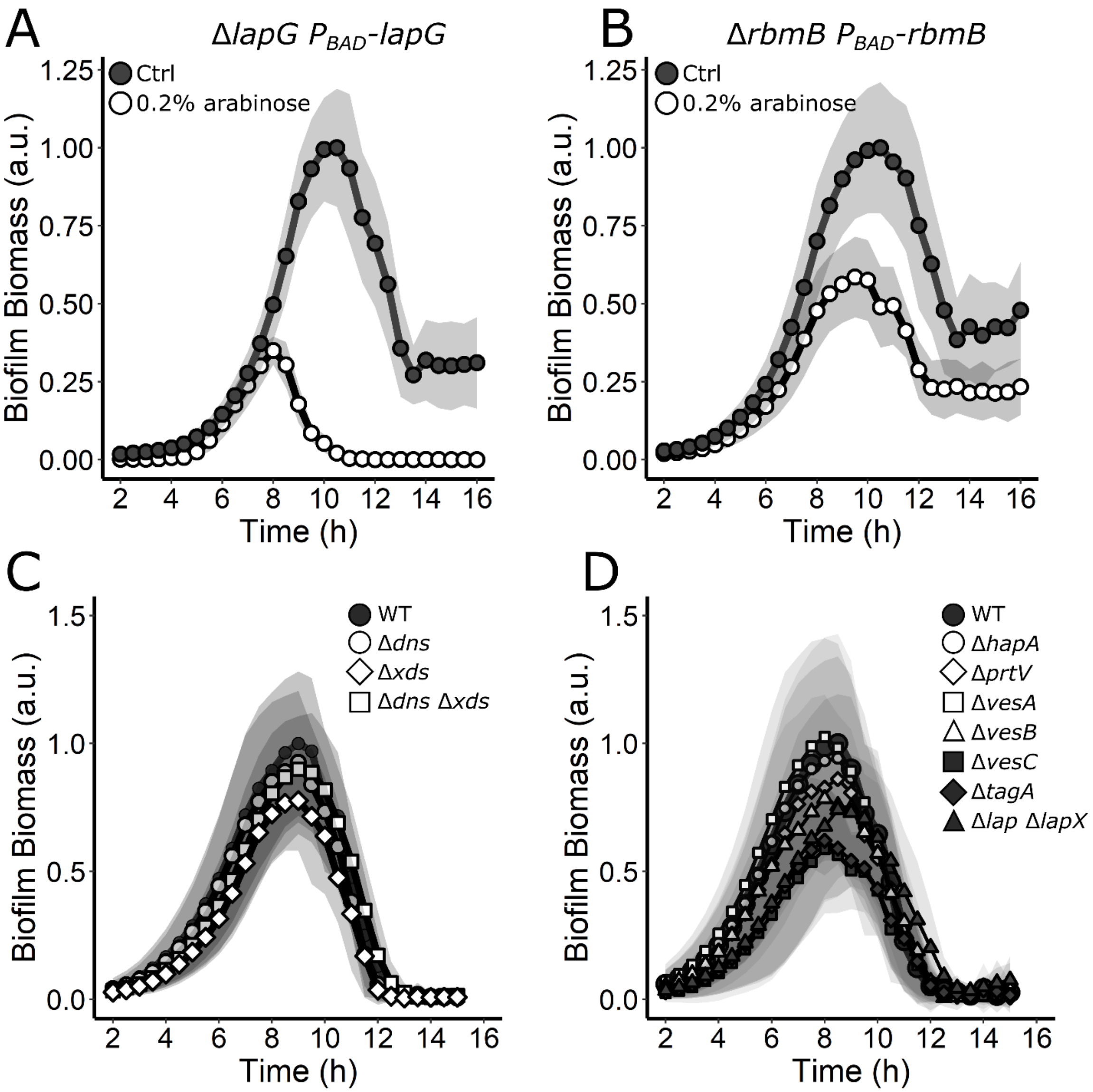

Fig. S3. Introduction of *lapG* and *rbmB* complements the Δ*lapG* and Δ*rbmB* biofilm defects, respectively, and assessment of the roles of extracellular DNAses and secreted proteases in *V. cholerae* biofilm dispersal. (A) Quantitation of biofilm biomass over time measured by time-lapse microscopy for the Δ*lapG* P_{BAD}-*lapG* strain following addition of water (Ctrl) or 0.2% arabinose. (B) As in A, but for the Δ*rbmB* P_{BAD}-*rbmB* strain. (C) Quantitation of biofilm biomass over time measured by time-lapse microscopy for WT *V. cholerae* and mutants lacking the designated DNAses. (D) Quantitation of biofilm biomass over time measured by time-lapse microscopy for WT *V. cholerae* and mutants lacking the designated proteases. In all cases, *N* = 3 biological and *N* = 3 technical replicates, ± SD (shaded). a.u., arbitrary unit.

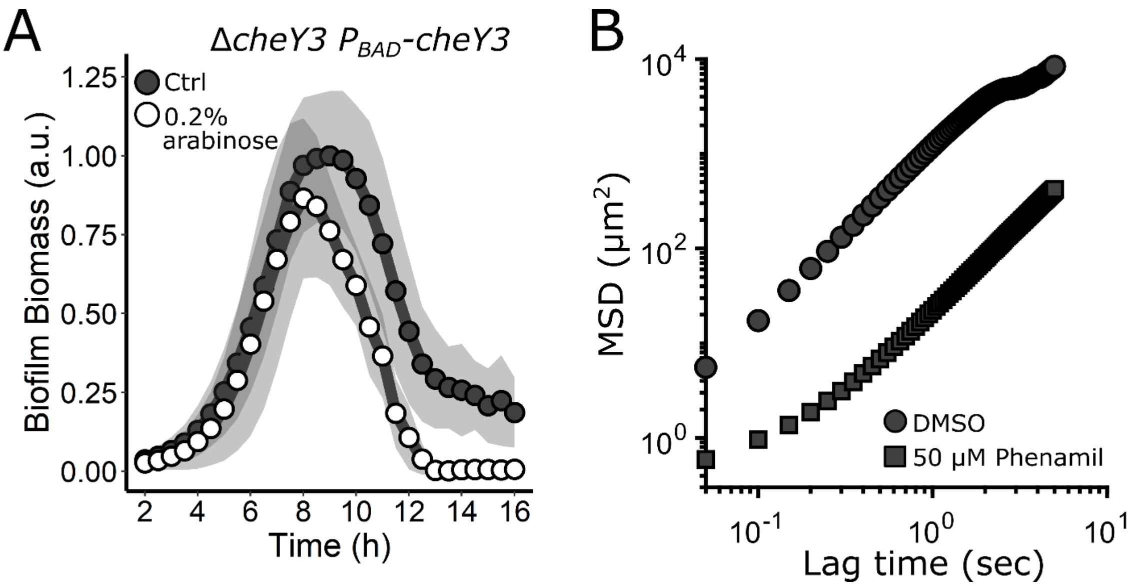

Fig. S4. Complementation of the Δ*cheY3* mutant and phenamil inhibition of *V. cholerae* motility. (A) Quantitation of biofilm biomass over time measured by time-lapse microscopy for the Δ*cheY3* P_{BAD}-*cheY3* strain following addition of water (Ctrl) or 0.2% arabinose. In all cases, *N* = 3 biological and *N* = 3 technical replicates, ± SD (shaded). a.u., arbitrary unit. (B) Mean squared displacement (MSD) of cell trajectories versus lag time for WT *V. cholerae* treated with DMSO solvent or 50 μM phenamil.

Table S1. Strains used in this study.

Strain Number	Genotype	Plasmid	Antibiotic Resistance	Parent
BB_Vc_0090	WT O1 El Tor biotype C6706str2	-	Sm	-
AB_Vc_761	Δ*vc1807*::Cm^R (Referred to as WT)	-	Sm, Cm	BB_Vc_0090
AB_Vc_705	Δ*cheY* Δ*vc1807*::Cm^R	-	Sm, Cm	BB_Vc_0090
AB_Vc_708	Δ*bipA* Δ*vc1807*::Cm^R	-	Sm, Cm	BB_Vc_0090
AB_Vc_839	Δ*mbaA* Δ*vc1807*::Cm^R	-	Sm, Cm	BB_Vc_0090
AB_Vc_711	Δ*potD1* Δ*vc1807*::Cm^R	-	Sm, Cm	BB_Vc_0090
AB_Vc_757	Δ*lapG* Δ*vc1807*::Cm^R	-	Sm, Cm	BB_Vc_0090
AB_Vc_758	Δ*rocS* Δ*vc1807*::Cm^R	-	Sm, Cm	BB_Vc_0090
AB_Vc_775	Δ*dbfS* Δ*vc1807*::Cm^R	-	Sm, Cm	BB_Vc_0090
AB_Vc_777	Δ*cdgI* Δ*vc1807*::Cm^R	-	Sm, Cm	BB_Vc_0090
AB_Vc_778	Δ*cdgG* Δ*vc1807*::Cm^R	-	Sm, Cm	BB_Vc_0090
AB_Vc_485	Δ*rbmB* Δ*vc1807*::Kan^R	-	Sm, Kan	BB_Vc_0090
AB_Vc_801	Δ*vc1807*::Kan^R	pEVS143-P_{vpsL}-*lux*::Cm^R	Sm, Cm, Kan	AB_Vc_479
AB_Vc_825	Δ*cheY* Δ*vc1807*::Kan^R	pEVS143-P_{vpsL}-*lux*::Cm^R	Sm, Cm, Kan	AB_Vc_705
AB_Vc_829	Δ*lapG* Δ*vc1807*::Kan^R	pEVS143-P_{vpsL}-*lux*::Cm^R	Sm, Cm, Kan	AB_Vc_757
AB_Vc_802	Δ*rbmB* Δ*vc1807*::Kan^R	pEVS143-P_{vpsL}-*lux*::Cm^R	Sm, Cm, Kan	AB_Vc_485
AB_Vc_815	Δ*dbfS* Δ*vc1807*::Kan^R	pEVS143-P_{vpsL}-*lux*::Cm^R	Sm, Cm, Kan	AB_Vc_775
AB_Vc_936	WT	pBBR1-P_{vpsR}-*lux*::Cm^R	Sm, Cm	BB_Vc_0090
AB_Vc_938	WT	pBBR1-P_{vpsT}-*lux*::Cm^R	Sm, Cm	BB_Vc_0090
AB_Vc_942	Δ*dbfS* Δ*vc1807*::Kan^R	pBBR1-P_{vpsR}-*lux*::Cm^R	Sm, Cm, Kan	AB_Vc_815
AB_Vc_944	Δ*dbfS* Δ*vc1807*::Kan^R	pBBR1-P_{vpsT}-*lux*::Cm^R	Sm, Cm, Kan	AB_Vc_815
AB_Vc_773	Δ*dbfR* Δ*vc1807*::Cm^R	-	Sm, Cm	BB_Vc_0090
AB_Vc_701	Δ*dbfRS* Δ*vc1807*::Cm^R	-	Sm, Cm	BB_Vc_0090
AB_Vc_788	$dbfR^{D51V}$ Δ*vc1807*::Cm^R	-	Sm, Cm	BB_Vc_0090
AB_Vc_891	$dbfR^{D51V}$ Δ*dbfS* Δ*vc1807*::Kan^R	-	Sm, Kan	BB_Vc_0090
AB_Vc_863	*dbfR-SNAP* Δ*dbfS* Δ*vc1807*::P_{BAD}-*dbfS*::$Spec^R$	-	Sm, Spec	BB_Vc_0090
AB_Vc_865	Δ*dbfS* Δ*vc1807*::P_{BAD}-*dbfS*::$Spec^R$	-	Sm, Spec	BB_Vc_0090
AB_Vc_879	*dbfR-SNAP* Δ*vc1807*::Kan^R	-	Sm, Kan	BB_Vc_0090
AB_Vc_881	$dbfR^{D51V}$-*SNAP* Δ*vc1807*::Kan^R	-	Sm, Kan	BB_Vc_0090
AB_Vc_859	Δ*lapG* Δ*vc1807*::P_{BAD}-*lapG*::$Spec^R$	-	Sm, Spec	AB_Vc_757
AB_Vc_898	Δ*lapD* Δ*vc1807*::Kan^R	-	Sm, Kan	BB_Vc_0090
AB_Vc_900	Δ*lapD* Δ*lapG* Δ*vc1807*::Kan^R	-	Sm, Kan	BB_Vc_0090
AB_Vc_948	Δ*lapG* Δ*rbmB* Δ*vc1807*::Kan^R	-	Sm, Kan	AB_Vc_182
AB_Vc_862	Δ*rbmB* Δ*vc1807*::P_{BAD}-*rbmB*::$Spec^R$	-	Sm, Spec	AB_Vc_485
BB_Vc_0252	Δ*dns*	-	Sm	BB_Vc_0090
BB_Vc_0253	Δ*xds*	-	Sm	BB_Vc_0090
BB_Vc_0254	Δ*dns* Δ*xds*	-	Sm	BB_Vc_0090
MJ_552	Δ*hapA* Δ*vc1807*::Kan^R	-	Sm, Kan	BB_Vc_0090

MJ_553	Δ*prtV* Δ*vc1807*::Kan^{R}	-	Sm, Kan	BB_Vc_0090
MJ_554	Δ*vesA* Δ*vc1807*::Kan^{R}	-	Sm, Kan	BB_Vc_0090
MJ_555	Δ*vesB* Δ*vc1807*::Kan^{R}	-	Sm, Kan	BB_Vc_0090
MJ_562	Δ*vesC* Δ*vc1807*::Kan^{R}	-	Sm, Kan	BB_Vc_0090
MJ_561	Δ*lap* Δ*lapX lacZ*::*Ptac-mKO* Δ*vc1807*::Kan^{R}	-	Sm, Kan	BB_Vc_0090
AB_Vc_792	Δ*tagA* Δ*vc1807*::Cm^{R}	-	Sm, Cm	BB_Vc_0090
AB_Vc_857	Δ*cheY* Δ*vc1807*::P_{BAD}-*cheY3*::Spec^{R}	-	Sm, Spec	AB_Vc_705
AB_Vc_715	*cheY*$^{D16K,\ Y109W}$ Δ*vc1807*::Kan^{R}	-	Sm, Kan	BB_Vc_0090
AB_Vc_732	Δ*vpsL* Δ*vc1807*::*Ptac-mScarletI::*Spec^{R}	-	Sm, Spec	BB_Vc_0090
AB_Vc_735	Δ*cheY* Δ*vpsL* Δ*vc1807*::*Ptac-mScarletI*::Spec^{R}	-	Sm, Spec	AB_Vc_705
AB_Vc_745	*cheY*$^{D16K,\ Y109W}$ Δ*vpsL* Δ*vc1807::Ptac-mScarletI*::Spec^{R}	-	Sm, Spec	AB_Vc_715

Table S2. DNA oligonucleotides and gene fragments used in this study.

Oligo #	Name	Purpose	Direction	5' to 3' Sequence
**551**	*cheY*_3000up	Cloning at *cheY3* locus	F	CAAGCGTTACAACTCGCAGCCTAG
**552**	*cheY*_3000down	Cloning at *cheY3* locus	R	CACAACCAGACCTACGCGCTGAC
**553**	*cheY*_100up	Cloning at *cheY3* locus	F	GGTGAGGTACTTGGAGTTAGTGAATCTC
**554**	*cheY*_100down	Cloning at *cheY3* locus	R	CACTGAAGCGCTCATCAATCTGAAAG
**555**	*cheY*_B	*cheY3* deletion	R	GAGCACCTTTTGCCGCAGCAAAAGCCTGAGTTTGAGATCAGT GATATTTAGTCATTCC
**556**	*cheY*_C	*cheY3* deletion	F	GGAATGACTAAATATCACTGATCTCAAACTCAGGCTTTTGCT GCGGCAAAAGGTGCTC
**561**	*cheY*_2700up	Cloning at *cheY3* locus	F	GATGACCGTGTCAGTTTGCAATCGAG
**562**	*cheY*_2700down	Cloning at *cheY3* locus	R	CTTCGGTGCTAACCAGTTTTGTAAG*TAGA*AC
**563**	*cheY*_up_R	Cloning at *cheY3* locus	R	GAGTTTGAGATCAGTGATATTTAGTCATTCCGAGTCC
**564**	*cheY*_down_R	Cloning at *cheY3* locus	R	GGCTTTTGCTGCGGCAAAAGGTGCTCTATTC
**566**	*cheY*_D16K_Y109 W	Gblock for introduction of *cheY3* point mutation	F	GTTAAGTTCTTGGACTCGGAATGACTAAATATCACTGATCTC AAACTCAGTGGAGGCAATTTTGAATAAAAACATGAAGATCCT TATTGTTGATAAGTTTTCAACAATGCGCCGAATCGTTAAAAA CCTACTTCGAGATCTGGGGTTCAATAACACGCAGGAAGCGGA CGATGGCCTAACGGCATTGCCTATGCTCAAGAAAGGTGATTT TGACTTTGTAGTCACAGACTGGAATATGCCCGGTATGCAAGG TATTGACTTGCTTAAAAATATCCGTGCCGACGAAGAACTGAA GCACCTGCCTGTACTAATGATCACAGCAGAAGCCAAACGTGA GCAAATCATCGAAGCCGCTCAAGCAGGCGTGAATGGTTGGAT CGTAAAACCATTTACCGCTGCTACGCTTAAAGAAAAAT*TAGA* CAAAATTTTTGAGCGTTTATAAGGCTTTTGCTGCGGCAAAAG GTGCTCTATTCACACGCGCAAAAG
**545**	*bipA*_3000up	Cloning at *bipA* locus	F	GCTGCGTGAGCAGTTGTAAATCGAG
**546**	*bipA*_3000down	Cloning at *bipA* locus	R	CAACGCTTTGTAGTTCGGGATTAGCATATA
**547**	*bipA*_100up	Cloning at *bipA* locus	F	GTCGACGATTTCAGCGCGACAGATC
**548**	*bipA*_100down	Cloning at *bipA* locus	R	GAGGTATTTCTGGATAGGTGGCATAGC
**549**	*bipA*_B	*bipA* deletion	R	GATGACTTATCTTACCAAACGAAAGTCAGTGACGGGGTTTGC TTCACTTTTTCATTGAGGCTG
**550**	*bipA*_C	*bipA* deletion	F	CAGCCTCAATGAAAAAGTGAAGCAAACCCCGTCACTGACTTT CGTTTGGTAAGATAAGTCATC
**567**	*bipA*_2700up	Cloning at *bipA* locus	R	CAGTGACTCGTCCAAAATGAGCACTG
**568**	*bipA*_2700down	Cloning at *bipA* locus	R	GATCTAAATCGCCACTGATCCCATCAAG
**571**	*mbaA*_3000up	Cloning at *mbaA* locus	F	GCGCGCTAATCTGAACTCAACCCATAAG
**572**	*mbaA*_2700up	Cloning at *mbaA* locus	F	CGTTAGCATTCCACGCGGTCAGTTAG
**711**	*mbaA*_KO2_B	*mbaA* deletion	R	GGAGGCATGAAGCCATGGGGAGATCTCGCTATGGTTTAGCTT CATATTGGTAAGTCACACTG
**712**	*mbaA*_KO2_C	*mbaA* deletion	F	CAGTGTGACTTACCAATATGAAGCTAAACCATAGCGAGATCT CCCCATGGCTTCATGCCTCC
**575**	*mbaA*_2700down	Cloning at *mbaA* locus	R	GATCTCATGACGCGCCTGACGGTATTTAAG
**576**	*mbaA*_3000down	Cloning at *mbaA* locus	R	CATCGTTCGCGATAGTGGGAAATTCAATAAAATG
**577**	*mbaA*_100up	Cloning at *mbaA* locus	F	GAAACCTGACATTGCCGCAATCAATGC

**578**	*mbaA*_100down	Cloning at *mbaA* locus	R	CCTGCTTCCAATCCGACATAATACTCTGC
**539**	*potD1*_3000up	Cloning at *potD1* locus	F	CTGGAATCCGGTATGTGTGTGATGGTTAG
**540**	*potD1*_3000down	Cloning at *potD1* locus	R	AGAGCGACTAGGTGTTATTGAACTTGGG
**541**	*potD1*_100up	Cloning at *potD1* locus	F	CTAAGAAAAGCATCAAATAGGCAGCCATTG
**542**	*potD1*_100down	Cloning at *potD1* locus	R	GATCTGGAAGAGATTAAGGCGCTCTC
**543**	*potD1*_B	*potD1* deletion	R	GGTGGCTTTTTAATGGGAGATAAAAGGCTACGTTCCCATAGTGTA*TAGA*AAGAACC
**544**	*potD1*_C	*potD1* deletion	F	GGTTCTTTCTATACACTATGGGAACGTAGCCTTTTATCTCCCATTAAAAAGCCACC
**569**	*potD1*_2700up	Cloning at *potD1* locus	F	CTGATGATTATTGGTACGAGTTTTCTGACTCGTG
**570**	*potD1*_2700down	Cloning at *potD1* locus	R	CGATAATCCAAATCAAATCGAGGTGCAGG
**602**	*lapG*_3000up	Cloning at *lapG* locus	F	CAAACAATTACCCGGTTATTGGGGATG
**603**	*lapG*_2700up	Cloning at *lapG* locus	F	GCATTCCGTCAAAGTGCTCGATATTCATC
**604**	*lapG*_100up	Cloning at *lapG* locus	F	GATCATTCCGGGAATGACCGCTTC
**605**	*lapG*_B	Cloning at *lapG* locus	R	CGACTAGTTGTTTGTATAGCGTCATAGTGCAGGGCGGGCTATTCCCTCAGCGCATTGCTTTG
**606**	*lapG*_C	*lapG* deletion	F	CAAAGCAATGCGCTGAGGGAATAGCCCGCCCTGCACTATGACGCTATACAAACAACTAGTCG
**607**	*lapG*_100down	*lapG* deletion	R	GTGTTGTTGACTTCAGAGCGTTGTTG
**608**	*lapG*_2700down	Cloning at *lapG* locus	R	GTCCAGCCATTAACCAGATCAACAC
**609**	*lapG*_3000down	Cloning at *lapG* locus	R	CAGCGGTACTGGAATTGTCCTTGC
**774**	*lapD*_3000up	Cloning at *lapD* locus	F	CGCGAATACAAGAAGCGATCATGCAG
**775**	*lapD*_2700up	Cloning at *lapD* locus	F	GCAAACTTCTGCTTAAGCTCAAGATACTTGC
**776**	*lapD*_100up	Cloning at *lapD* locus	F	CAATTGGCTGGGGACTCTTCGAGAC
**777**	*lapD*_B	*lapD* deletion	R	GTATCTTGCATGCCTCTGACCTTGGAGTGCCTACTCATCATAGCTAAC
**778**	*lapD*_C	*lapD* deletion	F	GTTAGCTATGATGAGTAGGCACTCCAAGGTCAGAGGCATGCAAGATAC
**779**	*lapD*_100down	Cloning at *lapD* locus	R	GTAAGCCGTTGATCAGTGCTTCAGGAG
**780**	*lapD*_2700down	Cloning at *lapD* locus	R	CTAACTACGCGCAGTATGTTGAGTTACAAGCG
**781**	*lapD*_3000down	Cloning at *lapD* locus	R	CGTTCAAGCACAAGGCGATA*TAGA*CG
**784**	*lapDG*_B	*lapDG* deletion	R	GTATCTTGCATGCCTCTGACCTTGGAGGGCGGGCTATTCCCTCAGCGCATTG
**785**	*lapDG*_C	*lapDG* deletion	F	CAATGCGCTGAGGGAATAGCCCGCCCTCCAAGGTCAGAGGCATGCAAGATAC
**610**	*rocS*_3000up	Cloning at *rocS* locus	F	CAACTCGAGCTTTTCTACCAACCTCAG
**611**	*rocS*_2700up	Cloning at *rocS* locus	F	GCATTTTACCGCCCCATTTTCGC
**612**	*rocS*_100up	Cloning at *rocS* locus	F	CTTCAGGCCAAGATCCTTTTCTACTGTG
**613**	*rocS*_B	*rocS* deletion	R	GGTTTCCACCAATCAGAGTAAAATTAACCCCTTAAAATACTACCAACTGTCCGTGCGCGACGACG
**614**	*rocS*_C	*rocS* deletion	F	CGTCGTCGCGCACGGACAGTTGGTAGTATTTTAAGGGGTTAATTTTACTCTGATTGGTGGAAACC
**615**	*rocS*_100down	Cloning at *rocS* locus	R	GAAACCGATATAAACCGCATCGGCA

**616**	*rocS*_2700down	Cloning at *rocS* locus	R	GTCACGTTATTAGGCTTGGCGTATTTC
**617**	*rocS*_3000down	Cloning at *rocS* locus	R	GCTGTTTGTTCACCTTAGGCTCG
**533**	*vc1639*_3000up	Cloning at *dbfS* locus	F	GCTTAGTGATCGCAGAGCTTGC
**534**	*vc1639*_3000down	Cloning at *dbfS* locus	R	GTGCACTGCATTATTGACTCGCTTAGC
**535**	*vc1639*_100up	Cloning at *dbfS* locus	F	CAAGATTTTGACCGCGATTCCAATAC
**536**	*vc1639*_100down	Cloning at *dbfS* locus	R	G*TAGA*GTTTCCAAACCTATAGGAG
**626**	*vc1639*_Real_B	*dbfS* deletion	R	CAACTGAAAATCCGTTTTTGCACCGCATTTAATTGGCATGCAACTGATACCCAAG
**627**	*vc1639*_Real_C	*dbfS* deletion	F	CTTGGGTATCAGTTGCATGCCAATTAAATGCGGTGCAAAAACGGATTTTCAGTTG
**559**	*vc1639*_2700up	Cloning at *dbfS* locus	F	CAATCGGTGGTGCGCAACTTATCTGAG
**560**	*vc1639*_2700down	Cloning at *dbfS* locus	R	GTTAATGACTTGGAGCAGAATTAAGTTAGCCGC
**527**	*vc1638*_3000up	Cloning at *dbfR* locus	F	GTAGGTCTTCTCGCACTTGTGTTTTG
**528**	*vc1638*_3000down	Cloning at *dbfR* locus	R	GTCCATAACCTTAGCGGAACTCATG
**529**	*vc1638*_100up	Cloning at *dbfR* locus	F	GACAATCAAGTCTTTCGTGTCGAATACAAC
**530**	*vc1638*_100down	Cloning at *dbfR* locus	R	CTTCCAGCAAATATTGATGGATGAGATTTGGG
**628**	*vc1638*_Real_B	*dbfR* deletion	R	GAGATTTAATTGGCATGCAACTGATACCCAAGGTCTGCTCGATTATTTTTTGATGGCACG
**629**	*vc1638*_Real_C	*dbfR* deletion	F	CGTGCCATCAAAAAATAATCGAGCAGACCTTGGGTATCAGTTGCATGCCAATTAAATCTC
**557**	*vc1638*_2700up	Cloning at *dbfR* locus	F	CACCATCCGGTTTGTGCATCATGATG
**558**	*vc1638*_2700down	Cloning at *dbfR* locus	R	GTGGCGTCAGATCCCAAAACTTGTTC
**650**	*dbfR*_D51V_B	Generating *dbfR*^{D51V}	R	CAATTTCGGTAGGCCGAGTACGAGTACGATGACGTCC
**651**	*dbfR*_D51V_C	Generating *dbfR*^{D51V}	F	GGACGTCATCGTACTCGTACTCGGCCTACCGAAATTG
**736**	*dbfR*_SNAP_deltaS_Gblock	Gblock for generating *dbfR-SNAP* and simultaneously deleting *dbfS*	F	CGCGGTCTTGGGTATCAGTTGCATGCCAATTCAGGAAGCGGCTCAGGCAGCGGATCAGGAATGGATAAGGATTGTGAAATGAAGAGAACAACTT*TAGA*TTCCCCACTAGGTAAAT*TAGA*ATTATCCGGTTGCGAACAAGGATTACATCGTATTATATTTTTAGGAAAAGGAACCAGTGCAGCAGACGCCG*TAGA*AGTACCAGCCCCCGCCGCAGTTTTAGGAGGACCAGAACCACTAATGCAAGCCACCGCTTGGTTAAACGCATATTTTCATCAACCAGAAGCCA*TAGA*AGAATTCCCAGTACCAGCCCTACACCACCCAGTATTTCAACAAGAATCATTTACGAGACAAGTATTATGGAAATTATTAAAAGTCGTCAAATTCGGAGAAGTTATCAGCTATAGTCACCTAGCCGCTCTTGCCGGTAATCCAGCAGCCACTGCCGCAGTTAAAACCGCATTATCAGGTAACCCAGTTCCCATATTAATTCCATGCCA*TAGA*GTAGTACAAGGAGATT*TAGA*CGTCGGCGGATATGAAGGAGGTTTAGCAGTTAAAGAATGGTTACTAGCACATGAAGGACA*TAGA*TTAGGTAAACCAGGATTAGGTTAAATGCGGTGCAAAAACGGATTTTCAGTTGC
**734**	*dbfR*_R	Generating *dbfR*-SNAP and deleting *dbfS*	R	TTGGCATGCAACTGATACCCAAGACCGCG
**735**	*dbfS*_down_F	Generating *dbfR*-SNAP and deleting *dbfS*	F	ATGCGGTGCAAAAACGGATTTTCAGTTGC
**672**	SNAP_UnivR	Generating *dbfR*-SNAP	R	TTAACCTAATCCTGGTTTACCTAATCTATGTCCTTCATGTGCTAGTAACC
**718**	*dbfR*_SNAP_E	Generating *dbfR*-SNAP	F	GACA*TAGA*TTAGGTAAACCAGGATTAGGTTAAGATGTGATCAAAACTGTGCGCGGTC

**634**	*cdgI*_3000up	Cloning at *cdgI* locus	F	CGATGCAAGTAGCTGAACAAGCAC
**635**	*cdgI*_2700up	Cloning at *cdgI* locus	F	GAATACATTGACGCCGAGCGCTTTG
**636**	*cdgI*_100up	Cloning at *cdgI* locus	F	GGGAGCAACTTCACTGTATTCAATGAGTG
**637**	*cdgI*_B	*cdgI* deletion	R	GATGCGATCATCATGAGCTACCTATTTTTGTAAAGGCCCGAC TTCATTTTTTTCTACTCTC
**638**	*cdgI*_C	*cdgI* deletion	F	GAGAG*TAGA*AAAAAATGAAGTCGGGCCTTTACAAAAATAGGT AGCTCATGATGATCGCATC
**639**	*cdgI*_100down	Cloning at *cdgI* locus	R	GGTCAGCAGCTTTGCAGCACTTTATTG
**640**	*cdgI*_2700down	Cloning at *cdgI* locus	R	GAGGTGCAACCTGCGTGTAACTGGATTTTC
**641**	*cdgI*_3000down	Cloning at *cdgI* locus	R	CCAGTGAGGCTATCAATATGCGCATC
**642**	*cdgG*_3000up	Cloning at *cdgG* locus	F	GTGTCGATTCCAGCGACAAGTGCCAATTTG
**643**	*cdgG*_2700up	Cloning at *cdgG* locus	F	GAATACACCGCAGAGCCGATAGTGAC
**644**	*cdgG*_100up	Cloning at *cdgG* locus	F	GATAAATGCTGCCCAGTCGGCATAAACACTGAG
**645**	*cdgG*_B	*cdgG* deletion	R	GCACAAATTAATAGTTAATTAGCTTAAATATTAATCAGACTG GATAGTTGAGGATCAATCCTGATCC
**646**	*cdgG*_C	*cdgG* deletion	F	GGATCAGGATTGATCCTCAACTATCCAGTCTGATTAATATTTA AGCTAATTAACTATTAATTTGTGC
**647**	*cdgG*_100down	Cloning at *cdgG* locus	R	TTGAGGCCATGC*TAGA*GCATGATGTTGAGC
**648**	*cdgG*_2700down	Cloning at *cdgG* locus	R	CCAGTAAATTCGGGTTATGAGGTAAAGGATG
**649**	*cdgG*_3000down	Cloning at *cdgG* locus	R	GATCGCCACTTTCCGCGATTGGATG
**105**	BBC1881	Cloning at *vc1807* locus	F	TTTAAAGGGGATCAGTGACCG
**106**	BBC1882	Cloning at *vc1807* locus	R	CAATTTTGCTTTTGGACCATCCC
**270**	*1807*_2700up	Cloning at *vc1807* locus	F	GGCCGGCACTTTGATTACAATC
**271**	*1807*_2700down	Cloning at *vc1807* locus	R	GTCTATATCAGAGCGCTTAAAGAGCG
**721**	P_{BAD}_*1807*_Univ_B	Generating P_{BAD}-*dbfS*	R	CATTTCACACCTCCTGCAGGTAC
**722**	P_{BAD}-*dbfS-1807*_C	Generating P_{BAD}-*dbfS*	F	GTACCTGCAGGAGGTGTGAAATGGGTATCAGTTGCATGCCAA TTAAATCTCG
**723**	P_{BAD}-*dbfS-1807*_D	Generating P_{BAD}-*dbfS*	R	GTCGACGGATCCCCGGAATTTAATGGGATTTGACGGCTTTGG CTG
**232**	ABD123	Generating P_{BAD}-*dbfS*	F	ATTCCGGGGATCCGTCGAC
**729**	P_{BAD}-*lapG-1807*_C	Generating P_{BAD}-*lapG*	R	GTACCTGCAGGAGGTGTGAAATGAAACGTTGGATTGTGCTGT CTCTGG
**730**	P_{BAD}-*lapG-1807*_D	Generating P_{BAD}-*lapG*	F	GTCGACGGATCCCCGGAATCTACTCATCATAGCTAAC*TAGA*G G
**731**	P_{BAD}-*rbmB-1807*_C	Generating P_{BAD}-*rbmB*	R	GTACCTGCAGGAGGTGTGAAATGCTGTTATACTTAAATCAAT TCAATAAAGAGGGTGG
**732**	P_{BAD}-*rbmB-1807*_D	Generating P_{BAD}-*rbmB*	F	GTCGACGGATCCCCGGAATTCAATCTTTAATAAAGTGCTGTA TATAATAATGGTCGC
**724**	P_{BAD} -*cheY3-1807_Gblock*	Generating P_{BAD}-*cheY3*	F	GTACCTGCAGGAGGTGTGAAATGGAGGCAATTTTGAATAAA AACATGAAGATCCTTATTGTTGATGACTTTTCAACAATGCGC CGAATCGTTAAAAACCTACTTCGAGATCTGGGGTTCAATAAC ACGCAGGAAGCGGACGATGGCCTAACGGCATTGCCTATGCTC AAGAAAGGTGATTTTGACTTTGTAGTCACAGACTGGAATATG CCCGGTATGCAAGGTATTGACTTGCTTAAAAATATCCGTGCC GACGAAGAACTGAAGCACCTGCCTGTACTAATGATCACAGCA GAAGCCAAACGTGAGCAAATCATCGAAGCCGCTCAAGCAGG CGTGAATGGTTACATCGTAAAACCATTTACCGCTGCTACGCT

				TAAAGAAAAATTAGACAAAATTTTTGAGCGTTTATAAATTCC GGGGATCCGTCGAC
**587**	*tagA*_3000up	Cloning at *tagA* locus	F	GGGCTGCAAGAACTGGATCTGCTAC
**588**	*tagA*_2700up	Cloning at *tagA* locus	F	GAGCAAAATTACAAGCTCGATCTTCAGCTAAG
**662**	*tagA*_103bpD_B	Removes first 103 codons of *tagA* including start	R	GTCAAATACTGGTCGTTACTGGATGTTGCATTCTTTAACAAA AAAATAAAGACAAGGGAAACGTATTG
**663**	*tagA*_103bpD_C	Removes first 103 codons of *tagA* including start	F	CAATACGTTTCCCTTGTCTTTATTTTTTTGTTAAAGAATGCAA CATCCAGTAACGACCAGTATTTGAC
**591**	*tagA*_2700down	Cloning at *tagA* locus	R	CCACCGAGGATACCATCCATCTTGATAATATG
**592**	*tagA*_3000down	Cloning at *tagA* locus	R	CTCTTGCCATCCATATGACATGATGTCTTTTG
**593**	*tagA*_100up	Cloning at *tagA* locus	F	GTGTGGCTTCATCCATTGACCTCCAATG
**594**	*tagA*_100down	Cloning at *tagA* locus	R	CCACTGCGAAATTAATTTTAGGATCAGCTTTAGC
**664**	*tagA*_150down	Cloning at *tagA* locus	R	GCAACCATACATCTTCCATTACTACCATAAGAG
**519**	Arbitrary Primer	Transposon localization	F	GGCCACGCGTCGACTAGTACNNNNNNNNNNAGAG
**520**	*Tn*5 specific Primer	Transposon localization	R	GAAGCCCTTAGAGCCTCTC
**521**	Arbitrary PCR cleanup	Transposon localization	F	AGGAACACTTAACGGCTGAC
**522**	Arbitrary PCR cleanup	Transposon localization	R	GGCCACGCGTCGACTAGTAC

Movie SI (separate file). Representative time-lapse images of WT *V. cholerae* undergoing biofilm dispersal. Also shown are the phenotypes of mutants identified in a screen for strains defective in dispersal. The mutant categories are designated by color: signal transduction, blue; matrix degradation, green; motility, red.

SI References

1. E. A. Groisman, The pleiotropic two-component regulatory system PhoP-PhoQ. *Journal of Bacteriology* **183**, 1835–1842 (2001).
2. L. R. Prost, M. E. Daley, M. W. Bader, R. E. Klevit, S. I. Miller, The PhoQ histidine kinases of *Salmonella* and *Pseudomonas* spp. are structurally and functionally different: evidence that pH and antimicrobial peptide sensing contribute to mammalian pathogenesis. *Mol Microbiol* **69**, 503–519 (2008).
